# Dr. Rutter

**Published:** 1840-01

**Authors:** 


### JOHN RUTTER, M.D., OF LIVERPOOL.

(*From Dr. Jeffrey's Address at the Meeting of the Provincial Medical Association.*)

Since our last anniversary meeting we have to lament the death of our highly-respected and venerable senior brother in Liverpool, Dr. John Rutter. He was an able, scientific, and honorable practitioner amongst us for about half a century, and no less conspicuous for the urbanity of his manners than by his anxiety to promote every scientific object in the profession, or indirectly connected with it; nor has he left any one more competent to supply his place, as our leader and guide, whether we look at his character as a consulting physician or as one always selected to preside over our medical meetings. He was by disparity in age removed far beyond all competition amongst us for the last ten years of his life; and previous to that period, his seniority was always modestly assumed and cheerfully conceded. Most of our public institutions bear testimony to his intellectual labours, amongst which may be mentioned his activity in conjunction with the late Mr. Richard Heber, Mr. Roscoe, Mr. Clarke, and others, (I believe the late Rev. Dr. Frodsham Hodgson, Principal of Brazenose College, Oxford, who was a native of Liverpool,) in the formation of the Athenæum Library, and his exertions in forming our first Botanic Garden, the catalogue of which he arranged and wrote. He was for thirteen years one of five physicians attached to the Dispensary, to which were then appended the sick wards or infirmary of the workhouse, the fever hospital, a pauper lunatic asylum, the gaol, blue-coat hospital, and other minor charities, now all under separate and distinct medical officers. The Liverpool Medical Library had only imperfectly existed for seventy years, and never, until his untiring efforts, did it meet with a suitable resting-place; it is now permanent within the walls of this the Medical Institution; to the arrangement, improvement, and stability of which establishment he devoted much of his time, and nobly contributed to the extent of one thousand pounds. He also has enriched the building by a bequest of minerals and books, valued at more than that amount, and he would, had he been spared, have done much more. Although neither his age nor his habits allowed him to attend and enjoy our anniversary meetings, he always took a very lively interest in the proceedings of the association; and I frequently conversed with him upon the subject of the honour to be conferred upon us by the present reunion, from which he anticipated much satisfaction; and expressed an anxious wish that the medical meetings should be held within this building, the erection of which must be ascribed to his patronage. Dr. Rutter was a native of this town: he was a faithful member of that intellectual body, the Society of Friends; and he died at the age of 76, in affluence, the just reward of a well-spent life. The numerous attendance of his medical friends at his funeral amply evinced their respect and esteem; and I rejoice in having such an occasion to record my individual and personal experience of his abilities and uniform benevolence.

